# COX5B-Mediated Bioenergetic Alterations Modulate Cell Growth and Anticancer Drug Susceptibility by Orchestrating Claudin-2 Expression in Colorectal Cancers

**DOI:** 10.3390/biomedicines10010060

**Published:** 2021-12-28

**Authors:** Yu-De Chu, Siew-Na Lim, Chau-Ting Yeh, Wey-Ran Lin

**Affiliations:** 1 Liver Research Center, Chang Gung Memorial Hospital, Taoyuan 333, Taiwan; yudechu@cgmh.org.tw; 2 Department of Neurology, Linkou Chang Gung Memorial Hospital, Taoyuan 333, Taiwan; siewna@cgmh.org.tw; 3 College of Medicine, Chang Gung University, Taoyuan 333, Taiwan; 4 Molecular Medicine Research Center, Chang Gung University, Taoyuan 333, Taiwan; 5 Department of Hepatology and Gastroenterology, Linkou Chang Gung Memorial Hospital, Taoyuan 333, Taiwan

**Keywords:** cytochrome *c* oxidase subunit 5b, bioenergetic alteration, mitochondria, oxidative phosphorylation, glycolysis, Claudin-2, colorectal cancers

## Abstract

Oxidative phosphorylation (OXPHOS) consists of four enzyme complexes and ATP synthase, and is crucial for maintaining physiological tissue and cell growth by supporting the main bioenergy pool. Cytochrome *c* oxidase (COX) has been implicated as a primary regulatory site of OXPHOS. Recently, COX subunit 5B (COX5B) emerged as a potential biomarker associated with unfavorable prognosis by modulating cell behaviors in specific cancer types. However, its molecular mechanism remains unclear, particularly in colorectal cancers (CRCs). To understand the role of COX5B in CRCs, the expression and postoperative outcome associations using independent in-house patient cohorts were evaluated. A higher COX5B tumor/nontumor expression ratio was associated with unfavorable clinical outcomes (*p* = 0.001 and 0.011 for overall and disease-free survival, respectively. In cell-based experiments, the silencing of COX5B repressed cell growth and enhanced the susceptibility of CRCs cells to anticancer drugs. Finally, downstream effectors identified by RNA sequencing followed by RT-qPCR and functional compensation experiments revealed that the tight junction protein Claudin-2 (CLDN2) acts downstream of COX5B-mediated bioenergetic alterations in controlling cell growth and the sensitivity to anticancer drugs in CRCs cells. In conclusion, it was found that COX5B promoted cell growth and attenuated anticancer drugs susceptibility in CRCs cells by orchestrating CLDN2 expression, which may contribute to unfavorable postoperative outcomes of patients with CRCs.

## 1. Introduction

Colorectal cancers (CRCs) are among the most common malignancies worldwide as well as in Taiwan. The majority of CRCs are developed from adenomatous polyps named as the adenoma–carcinoma sequence, which eventually leads to metastasis [1]. In recent decades, bioenergetic alterations have been implicated in numerous types of cancers [2], including in CRCs [3,4,5], through their capability to control tumorigenesis and/or progression, thereby emerging as potent targets for anti-cancer treatments [6,7,8].

Bioenergy originates from anaerobic lactate fermentation and aerobic glycolysis as well as mitochondrial oxidative phosphorylation (OXPHOS) [9]. In normal cells under aerobic conditions, bioenergy is mainly generated through OXPHOS. Nearly a century ago, Otto Warburg discovered that cancer cells secrete more lactate than normal cells under aerobic conditions, indicating their higher glucose usage [10]. Although impaired mitochondrial metabolism and increased glycolysis have been observed in CRCs, it is evident that in addition to aerobic glycolysis, OXPHOS plays crucial roles in carcinogenesis/progression, implying the involvement of bioenergetic alterations in CRCs carcinogenesis/progression [3,4,5,11]. Thus, OXPHOS may serve as a novel target in cancer therapy [12,13,14,15].

OXPHOS is the main source of bioenergy supporting the physiological needs of cell growth and progress of intracellular metabolic pathways [16]. OXPHOS is composed of five complexes: complex I (NADH: ubiquinone oxidoreductase), complex II (succinate dehydrogenase), complex III (cytochrome *bc*1 complex), complex IV (cytochrome *c* oxidase, COX), and complex V (ATP synthase). These complexes are localized within the inner mitochondrial membrane [16]. During the activation of OXPHOS, the pumping of protons by complexes I, III, and IV from the mitochondrial matrix to the inter-membrane space leads to an increase in the membrane potential across the inner mitochondrial membrane. Next, complex V actively drives the flow of protons back to the matrix, resulting in the generation of ATP from adenosine diphosphate [16]. Among these processes, COX (complex IV) has been suggested as a major regulatory site of OXPHOS [17,18]. Thirteen subunits, including three mitochondrial DNA- and ten nuclear DNA-encoded subunits, are required for full enzymatic activity of this macrocomplex. Six of these ten nuclear-encoded subunits can be replaced by isoforms, leading to heterogeneity in the composition and activity of this large complex [19]. Recently, defects or irregular expression of subunits in COX have been associated with tumorigenesis or progression in several types of cancers, including hepatocellular carcinoma (HCC), glioma, breast cancer, and CRCs [20,21,22,23]. However, the roles of the subunits are unclear; some are oncogenic, whereas others are suppressive in these cancers. 

Recently, two distinct reference datasets were comparatively analyzed, which revealed that several subunits of COX were significantly elevated or downregulated in HCC. Only COX subunit 5B (COX5B) harbored an oncogene-like property, as HCC patients with higher COX5B expression showed poorer clinical outcomes [24]. Moreover, a signaling cascade composed of AMPK-UHMK1-ERK operating through COX5B-mediated bioenergetic alterations was also demonstrated, and further reinforcing the hypothesis that COX5B has an oncogene-like property. However, its role in CRCs remains unknown to date. Therefore, the effects of COX5B expression in CRCs and the underlying molecular mechanisms were investigated in this study.

## 2. Materials and Methods

### 2.1. Patients and Samples

This study was conducted under the approval of the Institutional Review Board of Chang Gung Memorial Hospital, Taiwan [Approval number: 101-0102B (19 June 2012), 202001598B0 (4 October 2020), and 201900381A3 (23 April 2019)]. Three cohorts were enrolled in this study. In cohort-1, 126 pairs in total, non-tumorous and tumorous, of freshly acquired tissues resected from CRCs patients were employed for Western blot and Seahorse analyses. In cohort-2, a total of 268 paraffin-embedded tissue sections derived from CRCs patients were used for immunohistochemical staining. Their clinical parameters were retrospectively reviewed, including gender, age of CRCs diagnosed, height, weight, body mass index (BMI), tumor location, tumor differentiation status, local invasion stage, tumor size, carcinoembryonic antigen (CEA) level, adjuvant therapy after surgical resection, distance of tumor from serosa, date of CRCs diagnosed, date of death, and date of disease progression. A total of 64 patients with adenomatous polyps were included in the third cohort for Western blot and Seahorse analyses.

### 2.2. Western Blot

The Western blot was conducted as mentioned previously [25]. The rabbit monoclonal antibodies against COX5B (abcam, Cat. ab180136, 1:30000 dilution), CLDN2 (Cell Signaling, Cat. #48120, 1:2000 dilution), and ACTB (Sigma-Aldrich, St. Louis, MO, USA, Cat. A5441, 1:10000 dilution), and the rabbit polyclonal antibody against GDAP1 (Abclonal, Cat. A6601, 1:2000 dilution), and SerpinB8 (Abclonal, Woburn, MA, USA, Cat. A13039, 1:4000 dilution) were used for Western blot analysis in this study.

### 2.3. Immunohistochemical Staining (IHC)

The IHC was conducted as procedures reported previously [26]. The rabbit monoclonal antibody against COX5B (abcam, Cambridge, UK, Cat. ab180136), and rabbit polyclonal antibody against CLDN2 (Abclonal, Cat. A6560) were used for IHC staining in 1:200 dilution. The intensity of staining signals was acquired by Image J (National Institutes of Health, Bethesda, Maryland, USA, Fiji version) and used for subsequent analysis.

### 2.4. Seahorse Assay

Simultaneous measurement of oxidative consumption rate (OCR), indicator of OXPHOS activity and extracellular acidification rate (ECAR), indicator of glycolysis activity, in either patient-derived tissues or cultured cells was conducted using the Seahorse XF24 analyzer (Agilent, Santa Clara, CA, USA) as previously described [3]. OCR and ECAR were reported as absolute rates normalized by the concentration of proteins extracted from tissues (mMol/min/mg for OCR while mpH/min/mg for ECAR) or from cultured cells (pMol/min/μg for OCR while mpH/min/μg for ECAR).

### 2.5. Cell Culture and Transfection

HT-29 and WiDr were used and maintained, respectively, in RPMI-1640 and DMEM mediums under standardized culture conditions with 5% CO_2_ in a humidified 37 °C incubator in this study. Both of the cell lines were routinely examined for mycoplasma contamination. For knockdown of *COX5B*, the smart pool siRNAs (Dharmacon, Lafayette, CO, USA, M-013632), siRNA 1: CGACUGGGUUGGAGAGGGA, siRNA 2: GAGCACCUGCACUAAAUUA, siRNA 3: GGGACUGGACCCAUACAAU and siRNA 4: GAGAA UAGUAGGCUGCAUC, were used. The non-targeting pool included four independent scramble siRNAs, UGGUUUACAUGUCGACUAA, UGGUUUACAUGUUGUGUGA, UGGUUUACAUGUUUUCUGA and UGGUUUACAUGUUUUCCUA, and was used as control siRNA (Dharmacon, D0018101020). The Lipofectamine RNAiMAX transfection reagent (Invitrogen, Waltham, MA, USA, 13778) was employed according to the protocol provided by the manufacturer. The plasmids capable of expressing COX5B and CLDN2 with myc-DDK tag were purchased from Origene (Rockville, MD, USA, Cat. RC202511 for COX5B and RC229728 for CLDN2). For transient expression of COX5B and/or CLDN2, the cells were seeded 16 h before transfection. Five μg of plasmid DNA was used for transfection in a 6 cm plate. The MaestroFectin (Omics Bio, New Taipei City, Taiwan, Cat. MF002) transfection reagent was used for transfection, according to the procedures provided by the manufacturer. 

### 2.6. Cytochrome c Oxidase (COX) Activity Measurement

To measure the activity of COX under changes to COX5B in CRCs cells, the cytochrome *c* oxidase assay kit (Sigma-Aldrich, Cat. CYTOCOX1) was employed. The mitochondrial extracts were isolated using a mitochondria isolation kit (Sigma-Aldrich, Cat. MITOISO2). All the experiments were conducted according to the instructions provided by the manufacturer. 

### 2.7. Cell Proliferation and Viability Assay

The cell proliferation assay was conducted as reported previously [27]. Briefly, 3 × 10^3^ cells were seeded in each 96-well plate. After at least 16 h post-seeding, the Alamar Blue cell viability reagent (Invitrogen, Cat. DAL1100) was directly supplemented to the culture medium 3 h before quantification of the fluorescence of the metabolite for day 1. Then, the quantification was measured every day until day 4. The cell viability in response to anticancer drugs was initiated from seeding 1 × 10^4^ cells in each well of a 96-well plate at least 16 h before treatments. The cells with 5-FU or Oxaliplatin supplemented were incubated at 37 °C incubator. After 24 h incubation, the medium of each well was refreshed with Alamar Blue cell viability reagent added. The quantification of the fluorescence of the metabolite was conducted after 3 h incubation at 37 °C. 

### 2.8. Transcriptomic Analysis

The transcriptomic profiling was conducted using RNA-sequencing (RNA-seq). A total of 2 μg total RNA was subjected into RNA-seq analysis. The RNA samples passed through the quality assessment by Agilent 2100 Bioanalyzer were then used for library construction according to the manufacturer’s instructions (Illumina, San Diego, CA, USA, Cat: 20020596). The sequencing was conducted using a NovaSeq 6000 system (Illumina) with a paired-end 150 bp program. The cleaned reads were aligned to human genome GRCh38 to obtain annotations for subsequent analysis of differentially expressed genes. 

### 2.9. Real-Time Quantitative Polymerase Chain Reaction (RT-qPCR)

The total RNA isolation was conducted as previously described [28]. The ToolScript MMLV RT kit (BIOTOOLS, New Taipei City, Taiwan, Cat. TGKRA04) was used for first strand cDNA synthesis for up to 5 μg total RNA. The QuantStudio 5 real-time PCR system was employed. The primers used in this study are listed in Table S1.

### 2.10. H_2_O_2_, Oligomycin, Antimycin, and NaN_3_ Treatment

To induce bioenergetic alterations, cells with around 80% confluence were treated with 1 μM Oligomycin (Cell Signaling Technology, Danvers, MA, USA, Cat. #9996), 2 μg/mL antimycin A (Sigma-Aldrich, Cat. A8674), or 5 mM NaN_3_ (Sigma-Aldrich, Cat. 71289) for 24 h. To accumulate ROS, cells were treated with 200 μM H_2_O_2_ (Sigma-Aldrich, Cat. 18304) for 16 h.

### 2.11. Statistical Analysis

Parametric data in normal distribution was presented as mean ± standard deviation and compared by Student’s *t*-test. Dichotomized data were presented as numbers and percentages (%) and compared by utilizing the Chi-square test. Univariate and following multivariate Cox proportional hazard models were performed to estimate survivals for clinical factors and other variables. In this study, significant factors determined from the univariate analysis could be included for multivariate Cox proportional hazards. The Kaplan–Meier method was performed to estimate the survival probabilities between groups, and the log-rank test was performed to compare the survivals. All tests were two-tailed, and *p* < 0.05 was considered statistically significant. All statistical analyses were performed using the Statistical Package for the Social Sciences (SPSS) statistics (IBM, Armonk, NY, USA, Version 20). 

## 3. Results

### 3.1. Higher COX5B Expression Levels in CRCs Correlate with Poorer Clinical Outcomes

**Figure 1:**
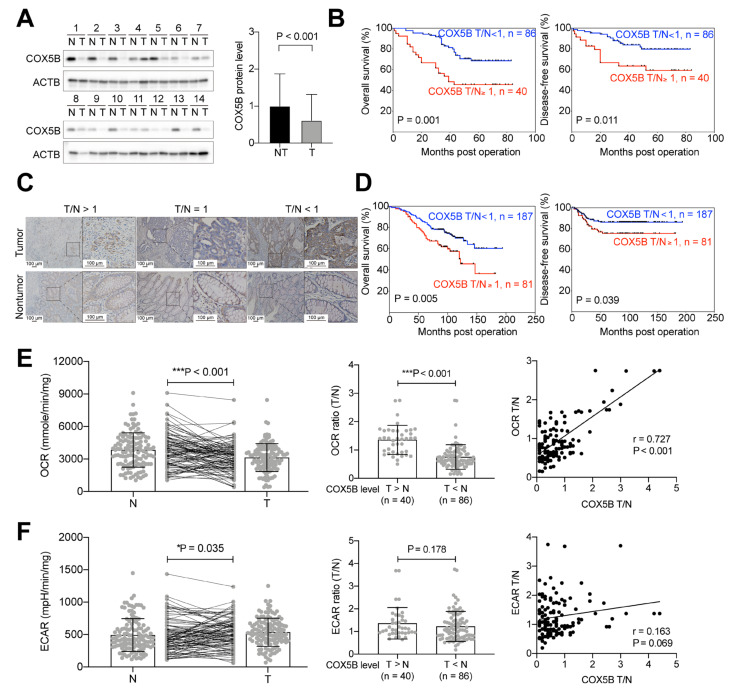
Increased COX5B is associated with unfavorable prognosis and elevated OCR in CRCs. (**A**) The representative images of Western blot analysis of lysates from both nontumorous (N or NT) and tumorous (T) tissues. COX5B, Cytochrome *c* Oxidase subunit 5B; ACTB, Actin Beta. The quantification results were shown in the right panel, analyzed by two-tailed paired Student’s *t*-test. (**B**) Kaplan–Meyer analysis of survival rates of subgroups of patients employed in (**A**). The subgroups of patients were separated according to the COX5B T/N ratio ≥ 1 or < 1 obtained from the Western blot analysis. (**C**) The representative images showing the IHC staining of COX5B on tissue slide sections. (**D**) Kaplan–Meyer analysis of survival rates of subgroups of patients employed in (**C**). The subgroups of patients were separated according to the COX5B T/N ratio ≥ 1 or < 1 obtained from the staining intensities of IHC. (**E**,**F**) The OCR and ECAR in either non-tumorous (N or NT) or CRCs tumorous (T) tissues. The *p* values in the left panels were acquired by two-tailed paired Student’s *t*-test, while two-tailed unpaired Student’s *t*-test was used for the middle panel. *, *p* < 0.05; ***, *p* < 0.001. Pearson correlation analysis was employed for the right panel.

To understand whether COX5B is involved in the oncogene-like properties of CRCs, its expression was assessed in patient-derived tissues. As shown in Figure 1A, unlike in HCC, the level of COX5B was significantly downregulated in tumorous tissues (*p* < 0.001). According to the ratio of tumorous/nontumorous (T/N ratio) of COX5B expression, approximately one-third of patients (40 of 126) exhibited COX5B T/N ≥ 1. Comparison of the baseline characteristics between subgroups with COX5B T/N < 1 and ≥ 1 are listed in Table S2. Patients with COX5B T/N ≥ 1 had greater height (*p* = 0.021), tumors located within the proximal colon (*p* = 0.007), tumors with good differentiation (*p* = 0.016), a shorter distance from the tumor to the serosa (*p* = 0.048), and a higher oxygen consumption rate OCR T/N ratio (*p* = 0.027).

To examine whether any clinicopathological parameter, COX5B expression, or bioenergetic alterations could predict overall survival (OS) and disease-free survival (DFS), Cox regression analysis was performed. As shown in Table 1, COX5B expression (*p* = 0.003), gender (*p* = 0.003), height (*p* = 0.013), tumor differentiation status (*p* = 0.029), local invasion grade (*p* = 0.025), tumor size (*p* = 0.006), circulating carcinoembryonic antigen (CEA) level (*p* < 0.001), OCR T/N ratio (*p* < 0.001), and extracellular acidification rate (ECAR) T/N ratio (*p* = 0.010) were associated with OS in univariate analysis. However, only COX5B expression (*p* = 0.011), gender (*p* = 0.025), circulating CEA level (*p* < 0.001), OCR T/N ratio (*p* = 0.015), and ECAR T/N ratio (*p* = 0.025) were independent predictors of OS in multivariate analysis. In contrast, the COX5B level (*p* = 0.002), gender (*p* = 0.021), age (*p* = 0.003), height (*p* = 0.007), tumor differentiation status (*p* < 0.001), local invasion grade (*p* = 0.012), tumor size (*p* = 0.014), circulating CEA level (*p* < 0.001), adjuvant chemotherapy received (*p* = 0.002), OCR T/N ratio (*p* < 0.001), and ECAR T/N ratio (*p* = 0.007) were associated with DFS. In multivariate analysis, the COX5B level (*p* = 0.004), gender (*p* = 0.023), age (*p* = 0.011), height (*p* = 0.023), tumor differentiation status (*p* = 0.005), adjuvant chemotherapy received (*p* = 0.011), and OCR T/N ratio (*p* < 0.001) remained as potential predictors of DFS. Kaplan–Meier analysis was conducted to confirm whether COX5B expression could predict the clinical outcomes of patients with CRCs. As shown in Figure 1B, patients with COX5B T/N ≥ 1 (n = 40) showed poorer OS (*p* = 0.001) and DFS (*p* = 0.011).

To confirm these findings, another cohort was evaluated. Paraffin-embedded tissue sections were retrieved for immunohistochemistry (IHC) staining of COX5B. As shown in Figure 1C, patients were classified into two groups: COX5B T/N < 1 and T/N ≥ 1 (T/N = 1 plus T/N > 1). Interestingly, similar to the results of Western blot analysis, approximately one-third of patients (81 of 268) exhibited COX5B T/N ≥ 1. Kaplan–Meier analysis of different subgroups further confirmed that patients with COX5B T/N ≥ 1 had poorer OS (*p* = 0.005) and DFS (*p* = 0.039) (Figure 1D).

Taken together, one-third of patients, those with higher COX5B expression in the tumorous area (COX5B T/N ≥ 1), exhibited associations with unfavorable clinical outcomes, suggesting that COX5B plays an oncogenic role in CRCs.

### 3.2. Higher COX5B Expression Levels Correlate with Increased OCR in CRCs

As a subunit of COX, a key modulator of OXPHOS, it was hypothesized that changes in COX5B expression disturbed bioenergetic homeostasis in CRCs. To test this hypothesis, the OCR and ECAR in the same cohort as shown in Figure 1A were measured in surgically resected tissues. As shown in Figure 1E left panel, the OCR was markedly decreased in the tumorous tissue (*p* < 0.001). Separating the patients into two groups according to the COX5B T/N ratio, T > N (n = 40) or T < N (n = 86), revealed an evident increase in the OCR T/N in those with higher COX5B T/N (Figure 1E middle panel, *p* < 0.001). Pearson correlation analysis also demonstrated that the COX5B T/N ratio was positively correlated with the OCR T/N in CRCs tissues (Figure 1E right panel, r = 0.727, *p* < 0.001).

In contrast, ECAR was significantly increased in the tumorous area (Figure 1F left panel, *p* = 0.035), whereas no obvious difference was observed between subgroups of patients with COX5B T > N and T < N (Figure 1F left panel, *p* = 0.178). In addition, COX5B T/N and ECAR T/N showed no significant correlation in tissues derived from patients with CRCs (Figure 1F right panel, r = 0.163, *p* = 0.069).

These results suggested that altered COX5B expression primarily disturbed OXPHOS activity but had only limited effects on promoting the activation of glycolysis in tissues from patients with CRCs. 

### 3.3. Higher COX5B Expression Levels Correlate with Increased OCR in the Pre-Cancerous Tissues

**Figure 2:**
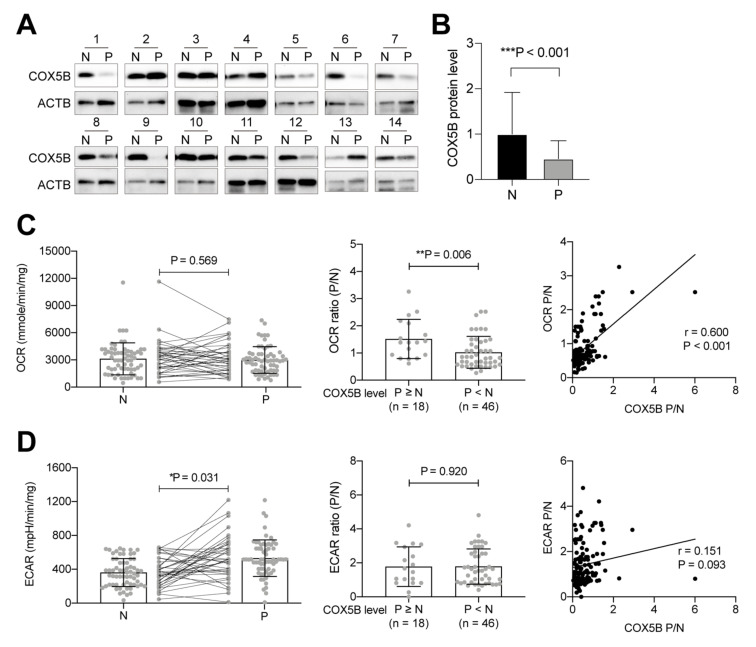
The increase of COX5B in adenomatous polyps is associated with elevated OCR. (**A**) The representative images of Western blot analysis of lysates from both normal mucosae (N) and polyps (P) tissues. COX5B, Cytochrome *c* Oxidase subunit 5B; ACTB, Actin Beta. The quantification results were shown in the (**B**), analyzed by two-tailed paired Student’s *t*-test. (**C**,**D**) The OCR and ECAR in either normal mucosa (N) and polyp (P) tissue. The *p* values in the left panels were acquired by two-tailed paired Student’s *t*-test, while two-tailed unpaired Student’s *t*-test was used for the middle panel. *, *p* < 0.05; **, *p* < 0.01; ***, *p* < 0.001. The Pearson correlation analysis was employed for the right panel.

To test whether elevated COX5B T/N is also correlated with a higher OCR T/N in the tissues under the pre-cancerous state of adenomatous polyps, the assays shown in Figure 1 were conducted. The expression status of COX5B was analyzed using Western blotting (Figure 2A); the quantitative results are shown in Figure 2B. As observed in CRCs, COX5B expression levels were significantly decreased in the tissues of adenomatous polyps (*p* < 0.001), suggesting that downregulation of COX5B occurred in tissues during the pre-cancerous state. 

To investigate the correlation between COX5B expression and OCR or ECAR in pre-cancerous tissues, freshly acquired tissues were obtained and subjected into Seahorse assays. As shown in Figure 2C left panel, there was no obvious change in the OCR between polyp and non-polyp tissues (*p* = 0.569). Among all patients, 18 of 64 (around one-third of all patients) showed a COX5B polyp/non-polyp (P/N) ratio ≥ 1, whereas the remaining 46 patients exhibited COX5B P/N < 1. Interestingly, a higher OCR P/N was associated with increased COX5B expression in adenomatous polyps (P/N ≥ 1) (Figure 2C middle panel, *p* = 0.006). Pearson correlation analysis also revealed a positive correlation between COX5B expression P/N and OCR P/N, indicating that higher COX5B expression is associated with elevated OCR in the polyps.

In contrast, ECAR was significantly increased in the polyps (Figure 2D left panel, *p* = 0.031). However, the COX5B P/N was not associated with the ECAR P/N (Figure 2D middle panel, *p* = 0.920). In addition, Pearson correlation analysis showed that COX5B P/N did not correlate with the ECAR P/N in tissues derived from patients with adenomatous polyps (Figure 2D right panel, r = 0.151, *p* = 0.093).

These findings suggest that alterations in COX5B expression and disturbance of OXPHOS activity observed in CRCs originated from the pre-cancerous state.

### 3.4. Change in COX5B Expression Influences Bioenergetic Alterations, Cell Growth, and Susceptibility to 5-Fluorouracil and Oxaliplatin in CRCs Cells

**Figure 3:**
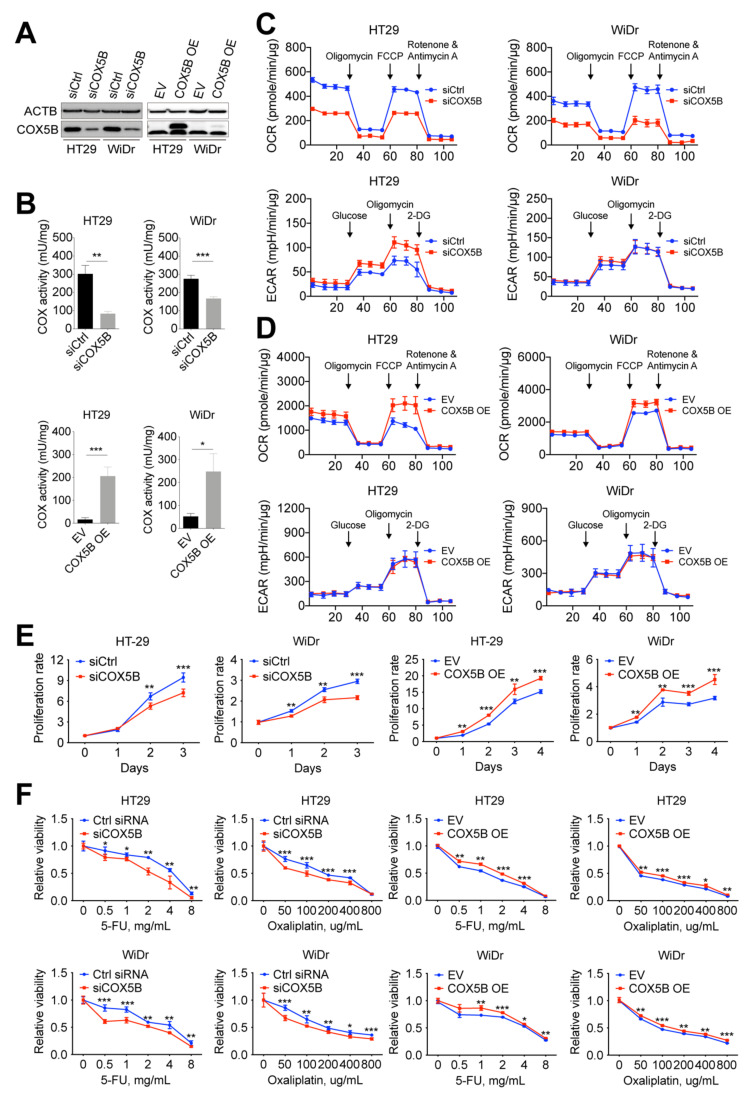
Change of COX5B expression affects bioenergetic alterations, cell growth, and sensitivity to anticancer drugs in CRCs cells. (**A**) The representative images of Western blot analysis. (**B**) The results of COX activity assay of CRCs cells with indicated treatments. COX, cytochrome *c* oxidase. The results of Seahorse assay of CRCs cells with silencing (**C**) or overexpressing COX5B (**D**). The proliferation ability (**E**) and sensitivity to anticancer drugs under indicated concentrations (**F**) in CRCs cells with either silencing or overexpressing COX5B were shown. *, *p* < 0.05; **, *p* < 0.01; ***, *p* < 0.001. All the *p* values were acquired by two-tailed paired Student’s *t*-test.

To investigate the impact of altering COX5B expression in CRCs, COX5B was knocked down or overexpressed in CRCs cells, including HT29 and WiDr cells (Figure 3A). As a crucial factor for COX, silencing of COX5B suppressed COX activity, whereas the gain-of-function of COX5B ameliorated COX activity in CRCs cells (Figure 3B). Furthermore, downregulation of COX5B significantly repressed OXPHOS activity but had only limited effects on modulating the glycolysis capacity (Figure 3C). In contrast, an increase in COX5B expression markedly elevated the OXPHOS capacity but did not change glycolysis activity (Figure 3D).

To determine the effects of alterations of COX5B expression in CRCs on cell growth, a cell proliferation assay was conducted. As was shown in Figure 3E, silencing of COX5B significantly attenuated the cell proliferation rate, whereas elevation of COX5B increased the cell renewal of CRCs cells. 

In addition, to examine whether alterations in COX5B expression affect the susceptibility of CRCs cells to the frequently employed anticancer drugs 5-fluorouracil (5-FU) and oxaliplatin, a drug sensitivity assay was conducted following silencing or upregulation of COX5B. As shown in Figure 3F, attenuating COX5B expression significantly sensitized CRCs cells to both 5-FU and oxaliplatin, whereas the opposite effects were observed in cells with elevated COX5B expression. Staining of the terminal deoxynucleotidyl transferase dUTP nick-end labeling (TUNEL)-positive cells supported these results (Figure S1).

Taken together, these findings partially reflect the findings in clinical patients showing that elevated COX5B expression levels led to bioenergetic alterations in CRCs and may modulate cell renewal and susceptibility to anticancer drugs, which may finally lead to recurrent disease and a poor prognosis.

### 3.5. COX5B-Mediated Bioenergetic Alterations Orchestrated Claudin-2 Expression in CRCs Cells

**Figure 4:**
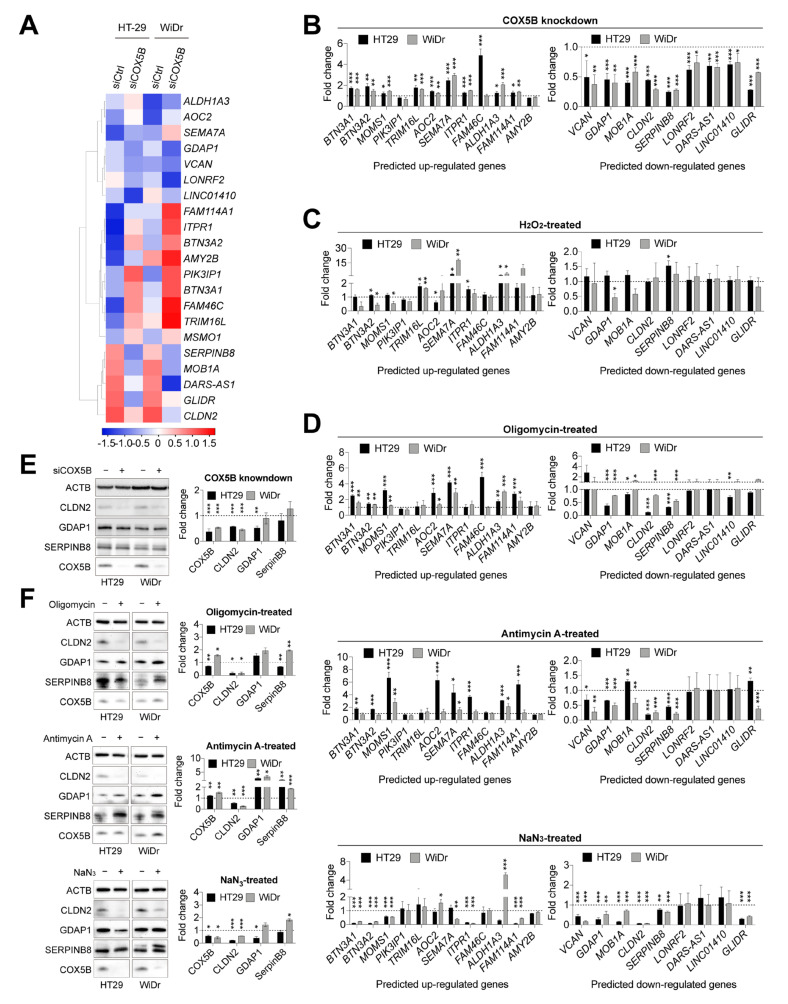
COX5B-mediated bioenergetic alterations orchestrates CLDN2 expression in CRCs cells. (**A**) The heat map representation of the relative expression of genes with log_2_-fold change > 0.5 and < −0.5 in cells with silencing COX5B, when compared to cells with scramble siRNA. The average linkage method was used for hierarchical clustering. The distances between rows and columns were computed by Pearson method. RT-qPCR was performed for the validation of indicated genes expression under (**B**) depletion of COX5B, (**C**) H_2_O_2_ treatment, and (**D**) treatment with OXPHOS inhibitors, including Oligomycin (upper), Antimycin A (middle), and NaN_3_ (lower). Western blot analysis was conducted for examination of indicated protein expression under (**E**) depletion of COX5B and (**F**) treatment with OXPHOS inhibitors, including Oligomycin (upper), Antimycin A (middle), and NaN_3_ (lower). −, cells without indicated treatment; +, cells with indicated treatment. *, *p* < 0.05; **, *p* < 0.01; ***, *p* < 0.001. All the *p* values were acquired by two-tailed paired Student’s *t*-test.

To investigate how COX5B modulates the aforementioned phenotypes in CRCs cells, searching for downstream effector of COX5B was conducted using transcriptomic analysis. As shown in Figure 4A, the heat map showed a set of gene candidates with significantly altered expression (log2-fold change < −0.5 or > 0.5). Confirmation experiments using real-time quantitative polymerase chain reactions (RT-qPCR) showed that the mRNA levels of *BTN3A1*, *BTN3A2*, *MOMS1*, *TRIM16L*, *AOC2*, *SEMA7A*, *ITPR1*, *ALDH1A3*, and *FAM114A1* were upregulated, whereas those of *VCAN*, *GDAP1*, *MOB1A*, *CLDN2*, *SERPINB8*, *LONRF2*, *DARS-AS1*, *LINC01410*, and *GLIDR* were downregulated in CRCs cell after the silencing of COX5B (Figure 4B).

As repressing COX5B expression may lead to either reduced OXPHOS activity or induced reactive oxygen species (ROS) accumulation (Figure S2), chemical drugs, including H_2_O_2_, oligomycin, antimycin A, and NaN_3_, were added into the growth medium to mimic the attenuation OXPHOS and accumulation of ROS to further understand the main source required for regulating the expression of these genes. As shown in Figure 4C, after treatment with H_2_O_2_, the mRNA levels of *TRIM16L*, *SEMA7A*, and *ALDH1A3* were upregulated, indicating that these genes were modulated by elevated ROS. However, none of the candidates predicted to be downregulated were reduced after treatment with H_2_O_2_.

Oligomycin, antimycin A, and NaN_3_ directly target components in OXPHOS and thus lead to bioenergetic alterations [12]. As shown in Figure 4D, the mRNA levels of only *GDAP1*, *CLDN2*, and *SERPINB8* consistently decreased after treatment with these OXPHOS-targeted chemical drugs, suggesting that these genes function downstream of COX5B-mediated bioenergetic alterations. 

To determine whether these changes also occurred at the protein level after silencing of COX5B and after treatment with these chemical drugs, the Western blot analysis of samples collected after the indicated treatments was conducted. Except for Claudin-2 (CLDN2), the protein levels of GDAP1 and SERPINB8 were not attenuated as predicted when COX5B was silenced (Figure 4E) or following treatment with OXPHOS-targeted chemical drugs (Figure 4F). Therefore, only the CLDN2 showed promise as a downstream protein of COX5B-mediated bioenergetic alterations. Notably, cells with NaN_3_-treated, which directly targets COX complex activity, showed significantly alleviated COX5B expression (Figure 4F).

Taken together, these findings indicate that expression of the tight junction localized protein CLDN2 [25] is a promising downstream effector orchestrated by COX5B-mediated bioenergetic alterations in CRCs cells.

### 3.6. Association of COX5B and CLDN2 Expression in Tissues from Patients with CRCs 

**Figure 5:**
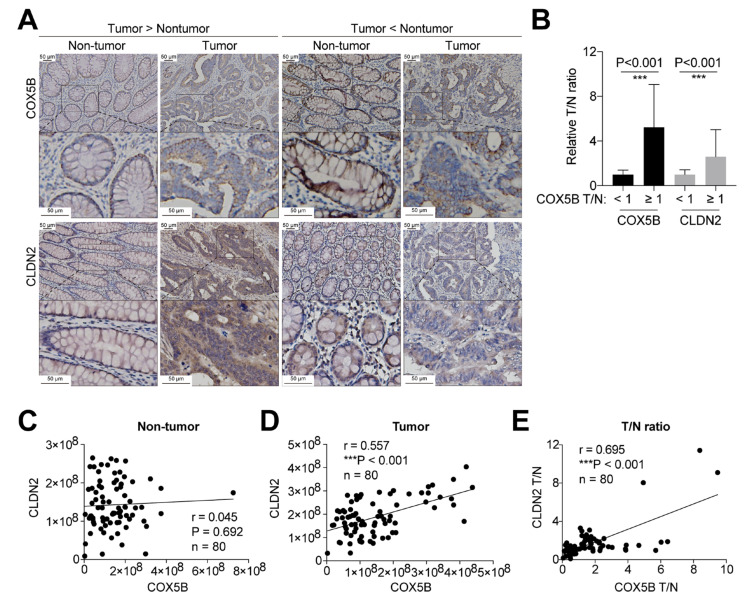
The correlation between COX5B and CLDN2 expression in CRCs patient-derived tissues. (**A**) The representative images of IHC staining of COX5B and CLDN2 in either non-tumor or tumor sections. The staining intensities were obtained and the comparisons between subgroups were summarized in (**B**). ***, *p* < 0.001. The Pearson correlation of COX5B and CLDN2 intensities in (**C**) non-tumor and (**D**) tumor sections were conducted. (**E**) The COX5B and CLDN2 T/N ratio were correlated using Pearson correlation analysis.

To assess whether the identified regulatory event involving CLDN2 downstream of COX5B-mediated bioenergetic alterations also occurred in samples derived from clinical patients with CRCs, IHC staining of both COX5B and CLDN2 on slide sections from these patients was conducted. As shown in Figure 5A, samples with a COX5B T/N ratio of staining intensities ≥ 1 were correlated with elevated CLDN2 T/N intensities, whereas the opposite results were observed in samples with COX5B T/N < 1. Comparisons of the COX5B and CLDN2 T/N ratios between subgroups with COX5B T/N < 1 and ≥ 1 are shown in Figure 5B. Further correlation of COX5B and CLDN2 staining intensities using Pearson correlation analysis revealed no correlation in the non-tumor sections (Figure 5C, r = 0.045, *p* = 0.692), but marked correlations in the tumor sections (Figure 5D, r = 0.557, *p* < 0.001). In addition, the COX5B and CLDN2 T/N ratios showed strong positive correlations (Figure 5E, r = 0.695, *p* < 0.001).

Taken together, these findings support that CLDN2 is a potent downstream effector of COX5B-mediated bioenergetic alterations in CRCs, because they showed significantly correlated expression patterns.

### 3.7. CLDN2 Functions Downstream of COX5B to Modulate Ell Growth and Susceptibility to 5-FU and Oxaliplatin

**Figure 6:**
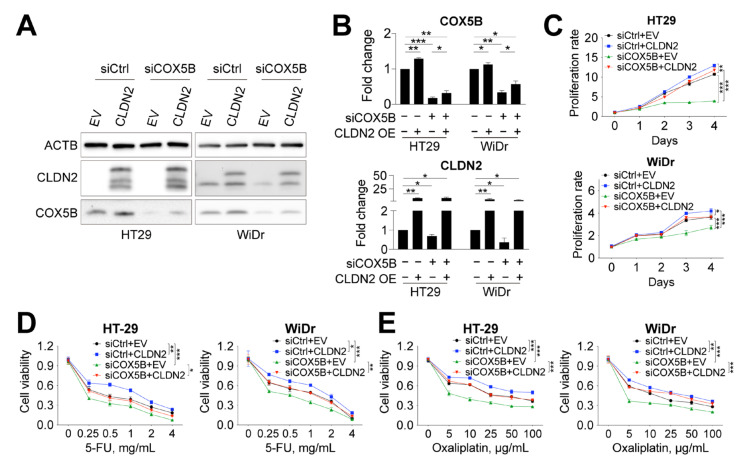
CLDN2 works downstream of COX5B-induced bioenergetic alterations in orchestration of cell growth and susceptibility to anticancer drugs in CRCs cells. (**A**) The representative images of Western blot analysis of lysates with indicated treatments. (**B**) The quantitative analysis and comparisons of protein levels of either COX5B and CLDN2 under indicated treatments. −, cells without indicated treatment; +, cells with indicated treatment. (**C**) The cell renewal capability was determined with or without COX5B silencing and CLDN2 overexpression in CRCs cells. The sensitivity of cells in response to the anticancer drugs, (**D**) 5-FU and (**E**) Oxaliplatin, was examined by cell viability assay. *, *p* < 0.05; **, *p* < 0.01; ***, *p* < 0.001. The *p* values in (**B**) were acquired by two-tailed paired Student’s *t*-test while in (**C**–**E**) were obtained by two-way ANOVA with correction by Bonferroni method.

To test whether CLDN2 act as a downstream effector of COX5B in controlling cell-based phenotypes (Figure 3E,F), CRCs cells with simultaneous silencing of COX5B and overexpression of CLDN2 (Figure 6A) were utilized to assess cell growth activity and the sensitivity to anticancer drugs. There was a positive feedback loop between COX5B and CLDN2, as overexpression of CLDN2 slightly enhanced COX5B expression, whereas silencing of COX5B repressed CLDN2 expression (Figure 6B). As shown in Figure 6C, overexpression of CLDN2 relieved the growth-suppressive effect caused by the silencing of COX5B in CRCs cells. Additionally, the susceptibility of CRCs cells to 5-FU and oxaliplatin was compensated when CLDN2 expression was elevated after silencing of COX5B (Figure 6D,E). The results of TUNEL staining also agreed with this notion (Figure S3).

These findings demonstrate that the tight junction protein CLDN2 serves as a downstream effector of COX5B-mediated bioenergetic alterations in orchestrating cell growth and susceptibility to anticancer drugs in CRCs cells.

## 4. Discussion

An increasing body of evidence from recent decades has supported that bioenergetic homeostasis is crucial for tumorigenesis and/or the progression of many cancer types, including HCC and CRCs [2,3,4,5,6,7,8,24]. COX5B has been reported as a growth-promoting gene that modulates downstream pathways in response to the induced bioenergetic alterations in HCC, breast cancer, and glioma, but its role in CRCs remains unknown [24,29,30,31]. As such, clinical associations and experimental assays were conducted to investigate the role and potential mechanism of action of COX5B in CRCs.

COX5B has also been implicated as a predictor of clinical outcomes in the various cancer types, including HCC, breast cancer, glioma, gastric cancer, head and neck squamous cell carcinoma (HNSCC), and clear cell renal cell carcinoma (ccRCC) [24,29,30,31,32]. In HNSCC, COX5B was reported as a potent tumor-suppressive gene, whereas in almost all other cancer types, this protein exhibited a growth-promoting property, and its higher levels were correlated with unfavorable clinical outcomes. Similar to these previous findings, our clinical association studies revealed that albeit COX5B was downregulated in around two-thirds of patients with CRCs, a higher COX5B T/N was associated with unfavorable clinical outcomes and an increased OCR T/N (Figure 1 and Table 1). Additionally, the correlation between COX5B expression and bioenergetic alterations could be traced to as early as the origin of the adenomatous–carcinoma sequence in the adenomatous polyps (Figure 2). Furthermore, increased COX5B expression in cells, a condition mimicking the situation found in one-third of patients with CRCs who had elevated COX5B expression in the tumorous tissue, promoted cell growth and reduced the susceptibility to anticancer drugs through orchestrating the COX-mediated bioenergetic alterations; experimentally reduced levels of COX5B, which simulated the situation observed in two-thirds of patients with CRCs who showed downregulated COX5B expression in tumorous tissue, repressed cell growth and sensitized CRCs cells to anticancer drugs (Figure 3). This evidence suggested that COX5B exerts a cancer-promoting role in CRCs, albeit its molecular mechanisms in modulating CRCs growth and susceptibility to anticancer drugs requires further investigation. 

Theoretically, in nontumorous tissues, aerobic OXPHOS, which requires sufficient expression of COX5B, is primarily used to generate cellular bioenergy, but is not involved in glycolysis under normal conditions [9]. As a well-established phenotype, it is considered that bioenergetic production sources shift from OXPHOS to aerobic glycolysis in many cancer types [10]. The downregulation of COX5B in tumorous tissues observed in approximately two-thirds of patients with CRCs may support this fact. A possible mechanism is that the status of hypoxic conditions modulates OXPHOS activity by regulating COX subunits expression, including COX5B, in mammalian cells [33,34]. HIF1A expression is increased and directly represses the expression of COX subunits by binding to the promoter region [33], indicating that COX5B expression is repressed under hypoxic conditions. This may explain why approximately two-thirds of patients with CRCs had reduced COX5B expression in the tumorous section, as the hypoxic status in tumors is well known. However, why one-third of patients with CRCs had unchanged or even elevated COX5B remains unclear. This result may be related to the level of oxygen transported into the tumors. A higher level of oxygen may help to maintain or even enhance COX5B expression in tumors. As described previously, angiogenesis occurring in tumors can promote tumor growth by providing nutrients, including oxygen [35]. The results of previous studies and our study suggest that tumors with low COX5B expression are possibly in an early stage or have not developed angiogenesis; in contrast, tumors with high expression of COX5B may be in a late stage or be undergoing angiogenesis, which may impact the unfavorable prognoses of patients with CRCs.

Analysis of the underlying growth-modulating and anticancer drugs sensitivity-controlling mechanisms of COX5B in CRCs revealed CLDN2 as a potential effector downstream of COX5B-mediated bioenergetic alterations (Figure 4). Subsequent experiments further confirmed this result (Figure 5), supporting that COX5B is positively correlated with CLDN2 expression in CRCs. CLDN2 is a promising tight junction localized protein with well-characterized functions, including promoting the tumorigenicity, metastasis, and susceptibility of cells to 5-FU, perhaps through ameliorating cancer stem cell property in CRCs [36,37,38]. Our findings partially reflect that CLDN2 acts as an effector downstream of COX5B-induced control of cell proliferation and sensitivity to anticancer drugs (Figure 6). Additionally, CLDN2 not only conferred resistance to 5-FU, but also contributed to reduction of the susceptibility to another frequently used anticancer drug, oxaliplatin, in CRCs. Interestingly, elevation of CLDN2 upregulated COX5B expression (Figure 6B), suggesting the presence of a positive feedback loop linking COX5B expression or bioenergetic homeostasis with CLDN2 levels in CRCs. This makes it worthy to be investigated in detail in the future.

## 5. Conclusions

In conclusion, we demonstrated that COX5B exerts oncogenic properties to ameliorate cell growth and repress susceptibility to anticancer drugs by modulating the expression of the oncogenic tight junction protein CLDN2 in CRCs, thereby contributing to the unfavorable prognostic outcomes of patients with elevated COX5B.

## Figures and Tables

**Table 1:** Cox proportional hazard analysis for association between clinical factors and OS or DFS.

Clinical Parameters	Univariate Analysis		Multivariate Analysis	
	HR (95% CI)	P	HR (95% CI)	P
*For OS*				
COX5B level, high expression = 1	2.451 (1.363–4.406)	0.003	2.424 (1.220–4.815)	0.011
Gender, male = 1	2.581 (1.368–4.867)	0.003	3.713 (1.182–11.664)	0.025
Age, per year increase	1.000 (0.974–1.027)	0.982		
Height, per cm increase	1.054 (1.011–1.098)	0.013	0.971 (0.911–1.036)	0.373
Weight, per kg increase	0.998 (0.968–1.029)	0.913		
BMI, per kg/m^2^ increase	0.918 (0.831–1.014)	0.091		
Tumor location, left-handed = 1	0.903 (0.486–1.679)	0.747		
Differentiation, poor = 1	2.483 (1.100–5.607)	0.029	1.234 (0.443–3.432)	0.688
Local invasion, per grade increase	1.702 (1.070–2.708)	0.025	1.707 (0.913–3.191)	0.094
Tumor size, per cm increase	1.003 (1.001–1.004)	0.006	1.001 (0.999–1.003)	0.196
CEA, per ng/mL increase	1.004 (1.002–1.005)	<0.001	1.004 (1.002–1.005)	<0.001
Adjuvant chemotherapy, yes = 1	1.129 (0.627–2.034)	0.686		
Distant to serosa, per mm increase	0.923 (0.845–1.008)	0.073		
OCR T/N, per number increase	2.676 (1.713–4.180)	<0.001	2.105 (1.157–3.830)	0.015
ECAR T/N, per number increase	1.610 (1.123–2.309)	0.010	1.683 (1.066–2.658)	0.025
*For DFS*				
COX5B level, high expression = 1	3.047 (1.503–6.177)	0.002	3.676 (1.514–8.927)	0.004
Gender, male = 1	2.439 (1.143–5.208)	0.021	8.162 (1.336–49.870)	0.023
Age, per year increase	0.953 (0.923–0.984)	0.003	0.938 (0.893–0.985)	0.011
Height, per cm increase	1.067 (1.018–1.118)	0.007	0.892 (0.809–0.984)	0.023
Weight, per kg increase	1.031 (1.000–1.064)	0.052		
BMI, per kg/m^2^ increase	1.019 (0.938–1.106)	0.663		
Tumor location, left-handed = 1	1.649 (0.710–3.830)	0.245		
Differentiation, poor = 1	4.547 (2.021–10.228)	<0.001	5.018 (1.619–15.547)	0.005
Local invasion, per grade increase	2.048 (1.171–3.583)	0.012	1.239 (0.581–2.642)	0.580
Tumor size, per cm increase	1.002 (1.000–1.004)	0.014	1.000 (0.998–1.002)	0.896
CEA, per ng/mL increase	1.003 (1.001–1.004)	<0.001	1.002 (1.000–1.004)	0.060
Adjuvant chemotherapy, yes = 1	4.158 (1.705–10.139)	0.002	4.186 (1.391–12.601)	0.011
Distant to serosa, per mm increase	0.895 (0.789–1.015)	0.083		
OCR T/N, per number increase	3.986 (2.428–6.546)	<0.001	4.005 (2.049–7.827)	<0.001
ECAR T/N, per number increase	1.753 (1.165–2.639)	0.007	1.512 (0.878–2.601)	0.136

Bold values indicate statistical significance *p* < 0.05. HR, Hazard ratio; BMI, body mass index; CEA, carcinoembryonic antigen; T/N, tumor/nontumor ratio. Distal colon (left-handed) includes rectum, sigmoid, descending and splenic flexure, while proximal colon (right-handed) contains transverse, hepatic flexure, ascending, and cecum.

## Data Availability

The data presented in this study are available on request from the corresponding author. The data are not publicly available due to the local Institutional Review Board regulation.

## Supplementary Materials

The following are available online at https://www.mdpi.com/article/10.3390/biomedicines10010060/s1. Figure S1: Silencing COX5B sensitized CRCs cells to anticancer drugs through enhancing programmed cell-death pathway; Figure S2: Silencing COX5B accumulates ROS in CRCs cells; Figure S3: CLDN2 works downstream of COX5B-induced bioenergetic alteration to modulate susceptibility to anticancer drugs in CRCs cells through enhancing programmed cell-death pathways. Table S1: Primer used in this study; Table S2: Baseline data for CRCs patients included in this study.
