# Supplementary material for: COX5B-Mediated Bioenergetic Alterations Modulate Cell Growth and Anticancer Drug Susceptibility by Orchestrating Claudin-2 Expression in Colorectal Cancers
Source: Biomedicines. 2021 Dec 28;10(1):60. doi: 10.3390/biomedicines10010060 (PMC8772867; doi:10.3390/biomedicines10010060)
Supplement: Supplementary file 1 [file biomedicines-10-00060-s001.zip › biomedicines-1514285-supplementary.pdf]

Supplementary information

Supplementary Figures

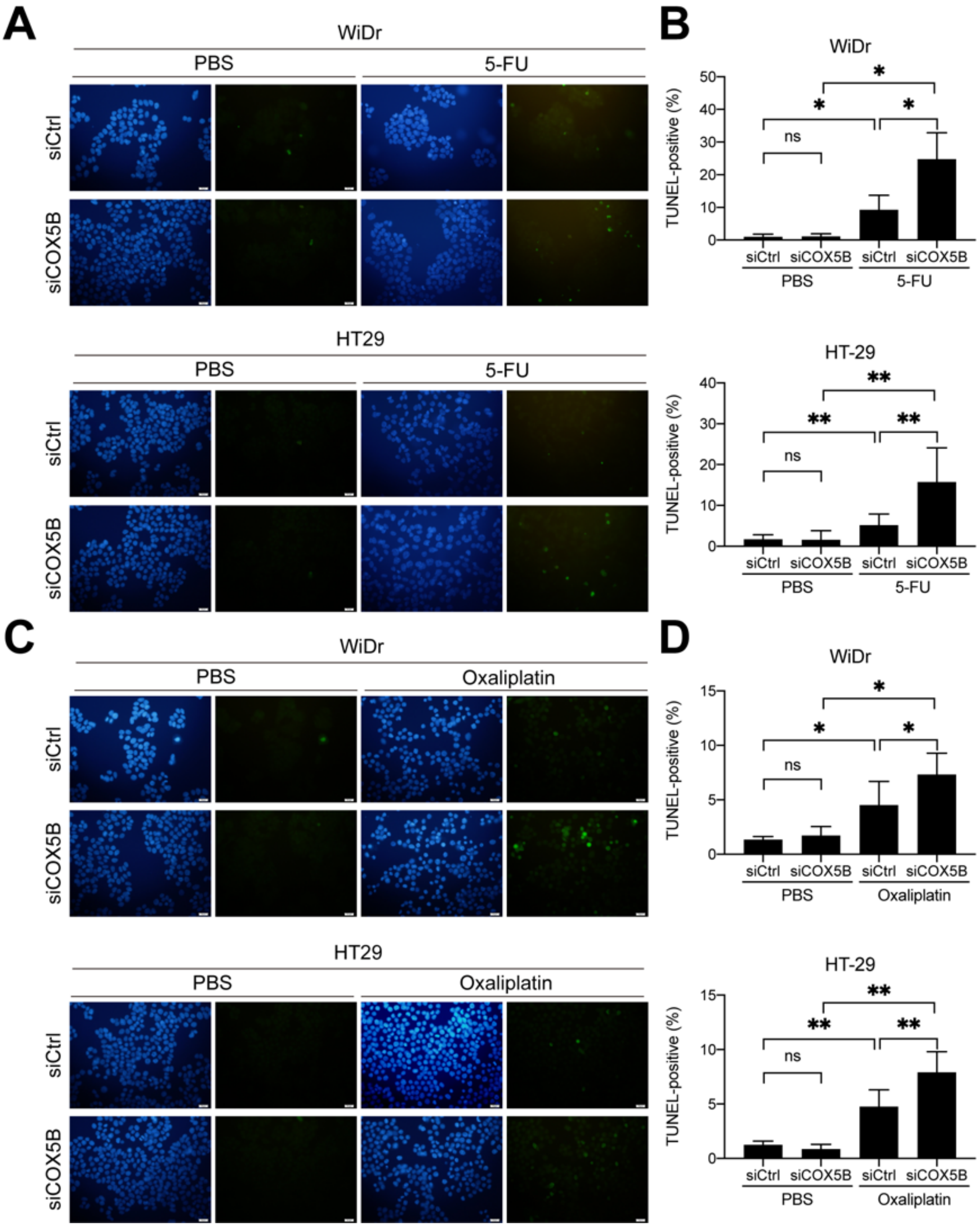

Figure S1. Silencing COX5B sensitized CRCs cells to anticancer drugs through enhancing programmed cell-death pathway. (A) The representative images showed the TUNEL-positive cells after with or without treatment of 1 mg/mL 5-FU. The quantification results are showed in (B). (C) The representative images showed the TUNEL-positive cells after with or without treatment of 100 mg/L Oxaliplatin. The

quantification results are showed in (D). *, P<0.05; **, P<0.01. All the P values were acquired by two-tailed paired student *t*-test.

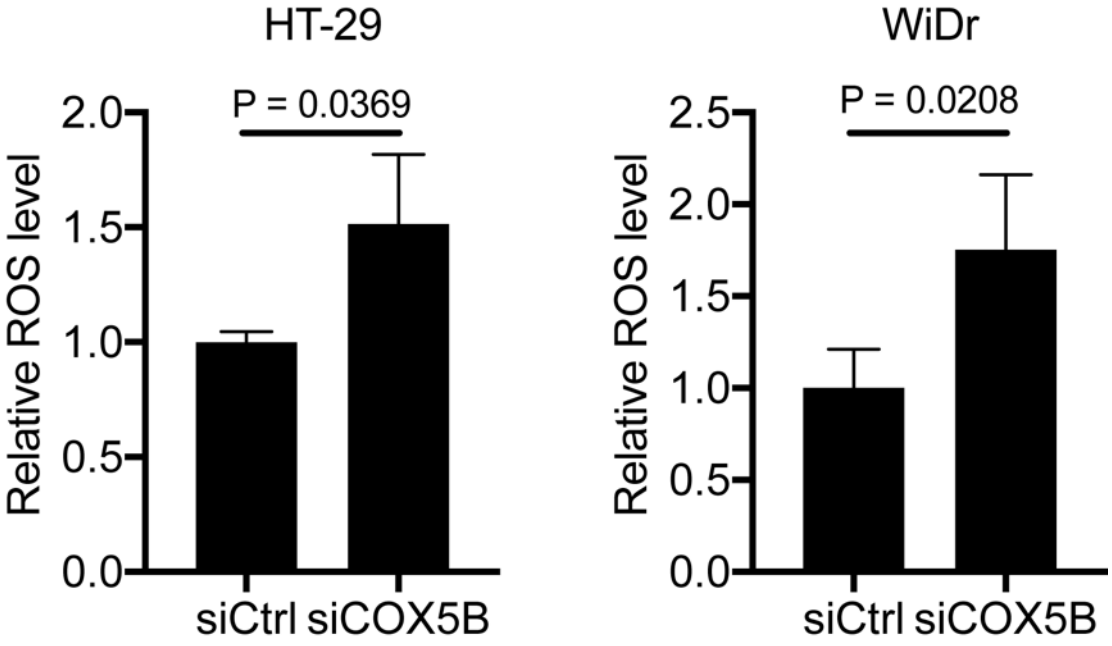

Figure S2. Silencing COX5B accumulates ROS in CRCs cells. The ROS in CRCs under depletion of COX5B was determined. The P values were acquired by two-tailed unpaired student *t*-test.

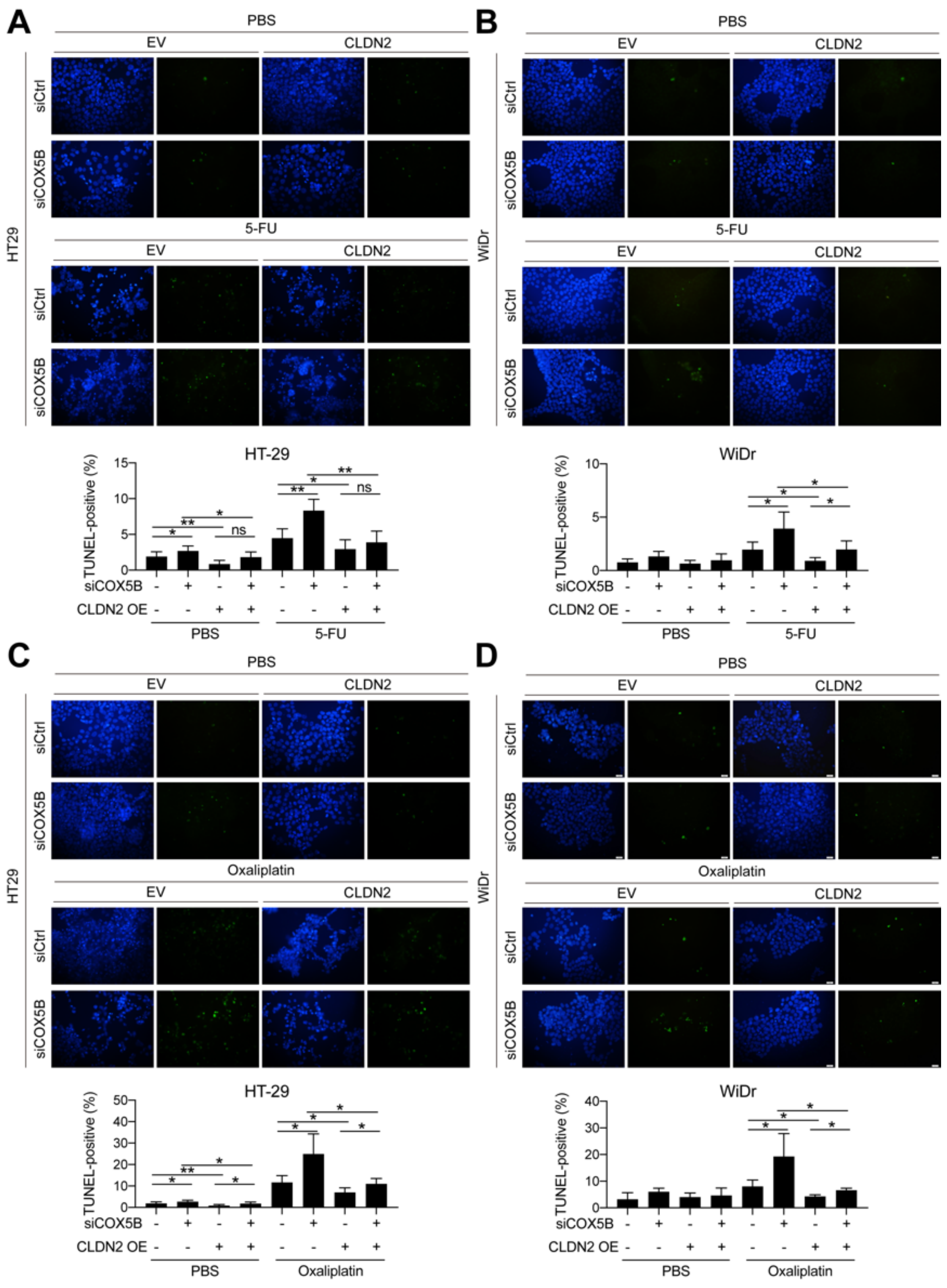

Figure S3. CLDN2 works downstream of COX5B-induced bioenergetic alteration to modulate susceptibility to anticancer drugs in CRCs cells through enhancing programmed cell-death pathway. (A and B) The representative images showed the TUNEL-positive cells under indicated treatments after with or without treatment of 1 mg/mL 5-FU. The quantification results are showed in the lower panels. (C and D) The

representative images showed the TUNEL-positive cells under indicated treatments after with or without treatment of 100 mg/L Oxaliplatin. The quantification results are showed in the lower panels. *, P<0.05; **, P<0.01; ns, no significance. All the P values were acquired by two-tailed paired student *t*-test.

Supplementary Tables

Table S1. Primers used in this study.

Name	Sequence (5'-3')
VCAN qPCR F	ACCACGCTTCCTATGTGAC
VCAN qPCR R	TGAACATCTTGGCCTTGGA
GDAP1 qPCR F	ACGACTTAAATCAAAGCTGC
GDAP1 qPCR R	TCCAAGACTTTCTCCAACTC
MOB1A qPCR F	TGGGAATCTGAGACAAGCTG
MOB1A qPCR R	AAGAAATCCACAGTGTTCACAG
CLDN2 qPCR F	GCAAGGTCTTCTAGATGCC
CLDN2 qPCR R	TGTAGCCCACAAGTTGGAG
SERPINB8 qPCR F	CGTGTGATTTCCTTCCAGAC
SERPINB8 qPCR R	GAAATCTTACCTTCAGTCTTCTCTG
LONRF2 qPCR F	TTAAAGGCTCAGGGTCACAG
LONRF2 qPCR R	AGATGAATTCTCCGAGCTCC
DARS-AS1 qPCR F	CAAAGCAAGACCCTGTGTC
DARS-AS1 qPCR R	ATTCCATGTCTTGGTCTCCC
LINC01410 qPCR F	GCTCTGCTCAGGATTTGTC
LINC01410 qPCR R	ACTAGCATCAACACCATCCA
GLIDR qPCR F	AGAAAGCTGAGACTCAGGG
GLIDR qPCR R	GTTTGGGATCGAGATCTGC
BTN3A1 qPCR F	CAACTTTCGTGTCTGCCTC
BTN3A1 qPCR R	ATGGTCTCTGCACTCATGG
BTN3A2 qPCR F	TGACAGTGGAAAGTACTTGTG
BTN3A2 qPCR R	GATTAGAACCCAGTGCTGC
MSMO1 qPCR F	CTGCATAGACTCTTACACCAC
MSMO1 qPCR R	CCATTCCAAATGGAGCCTG
PIK3IP1 qPCR F	ATCTGGAGGCTGTTTCTGG
PIK3IP1 qPCR R	CAGTAACTGTGATTGCCGG
TRIM16L qPCR F	GATGGAGAAGAGTAAGCAGG

TRIM16L qPCR R	AACTTGCAGTACTCCTCCA
AOC2 qPCR F	TCTTAGGAGAGGATCTGGTG
AOC2 qPCR R	TCACTGTGTTTGGGATGTC
SEMA7A qPCR F	GATACTGTCATGCAGAACCC
SEMA7A qPCR R	GTAGTAGATCTTGTCATCGTAAGC
ITPR1 qPCR F	TGCTTTATCTGTGGCTTGG
ITPR1 qPCR R	GTTGTGTTCTTCCTTGATGTG
FAM46C qPCR F	CTTCTATTGCCCAGTTTCCC
FAM46C qPCR R	GAAGGACATGCAATCCCTG
ALDH1A3 qPCR F	CTACTCTGAGTTTGTCAGGC
ALDH1A3 qPCR R	TTTGATCAATCTGAGGCCC
FAM114A1 qPCR F	TGACAAACTCAATAAGGCCA
FAM114A1 qPCR R	TTTGCCACATCTACTGACAC
AMY2B qPCR F	CTCCCAAGGGATTTGGAGG
AMY2B qPCR R	TGAAAGGGTTGTGAATTGCA
ACTB qPCR F	CACCAACTGGGACGACATGG
ACTB qPCR R	AGGATCTTCAGAGGTAGTC
COX5B qPCR F	GACCCATACAATGTACTG
COX5B qPCR R	CAGCCTACTATTCTCTTG

Table S2. Baseline data for CRCs patients included in this study.

Variable	Patients included for in assay		P
	COX5B T < N (n = 86)	COX5B T ≥ N (n = 40)	
Gender, male, n (%)	39 (45.3%)	24 (60.5%)	0.180
Age, years, mean ± SD	66.0 ± 11.0	63.0 ± 13.4	0.236
Height, cm, mean ± SD	156.7 ± 7.4	160.5 ± 8.4	**0.021**
Weight, kg, mean ± SD	58.6 ± 10.1	61.3 ± 8.8	0.214
BMI, mean ± SD	23.9 ± 4.1	23.4 ± 3.1	0.530
Tumor location, Left-handed, n (%)	67 (77.9%)	21 (52.5%)	**0.007**
Tumor differentiation, Well differentiation, n (%)	4 (4.7%)	8 (20.0%)	**0.016**
Local invasion, pT1 + pT2, n (%)	21 (24.4%)	8 (20.0%)	0.748
Maximum tumor size, mm, mean ± SD	20.1 ± 15.4	46.7 ± 173.7	0.766
CEA, ng/mL, median (range)	2.5 (0.5-505.5)	2.8 (0.5-1332.0)	0.375
Adjuvant chemotherapy, n (%)	42 (48.8%)	20 (50.0%)	0.903
Distance to serosa, mm, mean ± SD	4.2 ± 8.3	2.2 ± 3.6	**0.048**
OCR T/N ratio, mean ± SD	0.9 ± 0.5	1.1 ± 0.6	**0.027**
ECAR T/N ratio, mean ± SD	1.3 ± 0.7	1.2 ± 0.6	0.622

Bold values indicate statistical significance $P < 0.05$. BMI, body mass index; CEA, carcinoembryonic antigen. Distal colon (left-handed) includes rectum, sigmoid, descending and splenic flexure, while proximal colon (right-handed) contains transverse, hepatic flexure, ascending and cecum.
